# Iron Profile and Glycaemic Control in Patients with Type 2 Diabetes Mellitus

**DOI:** 10.3390/medsci4040022

**Published:** 2016-12-09

**Authors:** Gunjan Misra, Surendra Kumar Bhatter, Ajai Kumar, Varsha Gupta, Mohd Y. Khan

**Affiliations:** 1 Rheumatology Laboratory, Department of Biotechnology, Chhatrapati Shahu Ji Maharaj University, Kanpur 208024, India; gunjan.biotech@gmail.com; 2 Department of Biotechnology, Baba Saheb Bhimrao Ambedkar University, Lucknow 226025, India; profmykhan@rediffmail.com; 3 Internal Medicine Department, Regency Hospital, Sarvoday Nagar, Kanpur 208005, India; bhattersk@gmail.com; 4 Department of Biochemistry, Integral University, Lucknow 226026, India; ajay_sirbiochem@rediffmail.com

**Keywords:** T2DM, glycaemic control, serum Iron, Transferrin saturation, Kanpur, North India

## Abstract

Iron overload is increasingly being connected to insulin resistance in Type 2 Diabetes Mellitus (T2DM) patients. Free iron causes the assembly of reactive oxygen species that invariably steer the body’s homeostasis towards oxidative stress-mediated diabetic complications. This study aims to assess the serum iron, total iron binding capacity (TIBC), and percentage transferrin saturation (Tsat) of 150 subjects divided into three groups (I,II,III) of 50. Healthy individuals (controls) constituted Group I. Group II consisted of T2DM patients with optimal glycaemic control. T2DM patients with suboptimal glycaemic control formed group III. Mean serum free iron concentration was 105.34 ± 3.5, 107.33 ± 3.45, and 125.58 ± 3.45 μg/dL in Group I, Group II, and Group III, respectively. Mean serum TIBC concentration in Group I, Group II, and Group III was 311.39 ± 5.47, 309.63 ± 6.1, and 284.2 ± 3.18μg/dL, respectively. Mean serum transferrin saturation (%) in Group I, Group II, and Group III was 34.17 ± 1.21, 35.02 ± 1.2, and 44.39 ± 1.07, respectively. The difference between TIBC, mean serum free iron concentration, and transferrin saturation between Group I and Group III (for all, *p* values <0.001), as well as between Group II and Group III (*p* values 0.0012, 0.0015, and <0.0001, respectively) was statistically significant. The fasting plasma glucose values of Groups II and III were significantly higher than those of Group I, (*p* < 0.0001). Glycated haemoglobin (HbA1c) values were also shown to increase from Group I to II and then III, and the increase was highly significant (all *p* values <0.0001). Thus, decreased glycaemic control and an increase in the glycation of haemoglobin was the key to elevation in serum iron values and alterations in other parameters. However, a significant correlation was absent between serum iron and HbA1c (*r* = 0.05) and transferrin saturation (*r* = 0.0496) in Group III.

## 1. Introduction

The gigantic leap in the diabetic population is a serious health concern worldwide. By 2030, it is estimated that 79.4 million people will be in the grip of diabetes [1,2]. Middle and low income countries see more than 80% of the deaths owing to diabetes. It is further projected by the World Health Organization(WHO) that by 2030 the seventh leading cause of mortality in the world will be diabetes [3]. Furthermore, in developing countries, people in the age group of 40–60 are more prone to type 2 diabetes mellitus(T2DM), compared to older age groups in developed countries [4]. An Indian Council of Medical Research (ICMR)-funded study pointed out that the population affected by diabetes is lesser in north Indian states (Chandigarh 0.12 million, Jharkhand 0.96 million) as compared to Maharashtra (9.2 million) and Tamil Nadu (4.8 million) [5]. Incessant hyperglycaemia pooled with aberrations in the vital metabolic pathways (carbohydrate, fat, and protein) is the characteristic feature of T2DM. Unhinged insulin secretion and/or insulin action is the real culprit behind the chaos.

Mineral elements are not only integral members of the structural framework of body tissues, but are also inherently enveloped in various metabolic pathways. Their role is well documented in insulin yield, retention, and release, along with maintaining its configurational coherence [6]. Insulin resistance heralds the onset of type 2 diabetes, and various studies point to its connection with iron overload in the body [7,8]. Being a transition element, iron has marked redox activity, and any potential harm targeted at the body is prevented by its binding with transport or storage proteins [9]. In blood, iron is carried by transferrin; which is predominantly synthesized in the liver. In its free or non-transferrin-conjugated form, iron instigates the oxidation of biomolecules through Haber–Weiss and Fenton reactions via the generation of detrimental hydroxyl radicals [10]. These free radicals are powerful pro-oxidants which cause lysis of the lipid cellular membrane, damage the configurational harmony of proteins, and displace nucleic acids in genes [11,12,13]. Thus, the catalytic action of free iron is instrumental to insulin resistance in the beginning and later on to reduced insulin release, which subsequently results in the development of T2DM [14,15,16,17,18]. Emerging scientific evidence has disclosed that the relationship is bidirectional, wherein glucose metabolism also encroaches upon diverse iron-related pathways [19]. Oxidative stress-triggered inflammatory cytokines influence such alliances, replicating and reinforcing the initiated phenomenon [20]. Long-standing diabetic co-morbidities are also moderated by iron-mediated deterioration [21].

Inconsistent results have been reported for iron profile status in different studies across India. A study in Bangalore reported no significant difference in iron, transferrin, and total iron binding capacity (TIBC) values between diabetics and non-diabetics [22]. Similar results were seen in a Chennai study, where iron and TIBC values were not significant when compared between non-diabetics and diabetics (in both males and females) [8].Contrasting results were reported in another study, where an increase in the serum iron concentration of diabetics (with and without complications) was found to be significant when compared with controls [23]. Kanpur is one of the largest cities of north India, and to the best of our knowledge; no such study has been attempted in this region.

Hence, our study estimated the parameters associated with iron metabolism in relation to glycated haemoglobin (HbA1c) values in the selected population. The results of this study could serve as a basis for further studies in the region with larger cohort size.

## 2. Materials and Methods

The study was designed as a case–control study of diabetic patients visiting the outpatients department of government hospitals and some private clinics in Kanpur city. A total of 150 subjects were selected in the study, and based on their glycaemic control, they were sub-classified into three groups (I,II,III) of 50 each. Healthy individuals (controls) constituted Group I. Group II consisted of T2DM patients with optimal glycaemic control. T2DM patients with suboptimal glycaemic control formed Group III. The selection was random to minimize any bias.

### 2.1. Inclusion Criteria

The subjects selected for study were grouped as follows:

- Group I—Control group (*n* = 50), consisting of age- and sex-matched healthy subjects. They were free from any ailment which could affect the parameters under study. They were not on any medication, and were chosen from the general population.
- Group II—Diabetes mellitus type 2 patients with optimal glycaemic control (*n* = 50), disease duration less than 7 years and HbA1C) level less than 8%. They were on lifestyle modifications and oral hypoglycaemic drugs.
- Group III—Diabetes mellitus type 2 patients with suboptimal glycaemic control (*n* = 50), disease duration more than 7 years and HbA1C level more than 8%. They were on lifestyle modifications, oral hypoglycaemic drugs, insulin, or a combination of all three.

### 2.2. Exclusion Criteria

The exclusion criteria for patients extended to those diagnosed with type 1 DM, acute complications such as severe infections or major operations, trauma, severe cardiovascular/respiratory diseases, and pregnant and breast feeding women. Newly diagnosed cases and those suffering from chronic diabetic co-morbidities were also excluded from the study.

### 2.3. Methodology

Fasting venous blood was collected using standard clinical procedures. Height and weight were measured, and body mass index (BMI) was calculated as kg/m^2^.Blood glucose was measured by the glucose oxidase/peroxidase (GOD-POD) method, using a commercial kit from Span diagnostics (Surat, India), as previously described [24]. Glycated haemoglobin was measured by the modified colorimetric method of Fluckiger [25]. Iron and TIBC were estimated by using a commercial kit (Ferrozine method) produced by Coral Clinical systems (Goa, India). Briefly, iron bound to transferrin is released in an acidic medium, and the ferric ions are reduced to ferrous ions. The Fe (II) ions react with ferrozine to form a violet-coloured complex. The intensity of the formed complex is directly proportional to the amount of iron present in the sample. For TIBC, the serum is treated with an excess of Fe (II) to saturate the iron binding sites on transferrin. The excess Fe (II) is adsorbed and precipitated, and the iron content in the supernatant is measured to give the TIBC. Transferrin saturation (Tsat, %) is the ratio of serum iron and TIBC, and indicates how much serum iron is actually bound.

Transferrin saturation (%) = serum iron × 100/serum TIBC

All spectrometric readings were done using a UV-1800 spectrophotometer (Shimazdu, Kyoto Japan).

### 2.4. Ethical Statement

The study was approved by the institutional ethical committee of Ganesh Shankar Vidyarthi Memorial Medical College, Kanpur (G.S.V.M. Medical College, ethical code No.14/Steno, on 13 January 2011), and written informed consent was obtained from all the study subjects after explaining the objectives of the study. 

All possible precautionary measures were taken to prevent trace metal ion contamination (particularly iron) during all stages of the procedure. 

### 2.5. Statistical Analysis

All the values are expressed as Mean ± standard error of the mean (SEM), and a *p* value of <0.05 was considered statistically significant. Statistical significance of the differences between the mean values was analysed by one-way ANOVA followed by Tukey’s HSD post hoc test. Correlations of serum iron with other parameters were also studied by applying Pearson correlation test. Results were interpreted based on comparison between controls and diabetic subjects using SPSS software (version 15.0, Chicago, U.S.).

## 3. Results

Table 1 shows the general features of the subjects. The age difference between the subjects of all three groups (I, II, and III) was statistically not significant (all *p* values >0.05). The fasting plasma glucose (FPG) values of Groups II and III were significantly higher than those of Group I (*p* < 0.0001). However, between Groups II and III, the difference in FPG values was non-significant (*p* = 0.9448).

Glycated haemoglobin (HbA1c) values were shown to increase from Group I to II, and then III. This increase was highly significant (all *p* values < 0.0001).

Difference in BMI values between Group II and Group III subjects was not statistically significant (*p* = 0.826). However, their values were significant when compared with healthy controls of Group I (*p* = 0.0007 and *p* = 0.0051 respectively).

As shown in Table 2, mean serum free iron concentration in Group I, Group II, and Group III was 105.34 ± 3.5, 107.33 ± 3.45, and 125.58 ± 3.74 μg/dL, respectively, while mean serum TIBC concentration in Group I, Group II, and Group III was 311.39 ± 5.47, 309.63 ± 6.1, and 284.2 ± 3.18 μg/dL, respectively. Further, mean serum transferrin saturation (%) in Group I, Group II, and Group III was 34.17 ± 1.21, 35.02 ± 1.2, and 44.39 ± 1.07 μg/dL, respectively.

The difference between mean serum free iron concentration, TIBC, and Tsat between Group I and Group III (all three *p* < 0.05), as well as between Group II and Group III (*p* = 0.0012, 0.0015, and 0.0002, respectively) is highly statistically significant. However, the difference between these parameters in Group I and Group II is not significant (*p* = 0.9178, 0.9674, and 0.8622, respectively).

A significant correlation was absent between serum iron and HbA1c (*r* = 0.05) and Tsat (*r* = 0.0496) in diabetic patients of Group III (Table 3).

## 4. Discussion

The iron concentration values of Group II were statistically non-significant when compared with controls (Group I). On the contrary, the free iron concentration values of Group III were significantly higher, both when compared to Group I and Group II. This increase in iron levels may be explained in two different ways. Firstly, iron stores in the pancreas may lead to defective synthesis and secretion of insulin [26]. Secondly, excess iron deposition culminates in hyperinsulinemia due to obstruction in the insulin withdrawing ability of the liver [27]. Such deposits hinder insulin action, resulting in insulin resistance, which suppresses the yield of glucose in the liver [28].A similar trend has been observed in previous studies [29,30,31,32,33]. Poor glycaemic control is the root cause of escalated protein glycation—especially haemoglobin, which restores the free state of iron. This amplified free iron pool revitalizes oxidant generation, conferring damage to biomolecules and leading to complications [34].

Elevated transferrin saturation in the diabetic subjects of our study hints at ineffective erythropoiesis and accumulation of iron in human tissues, which hampers insulin action [19]. As demonstrated in three independent studies, transferrin saturation can act as an independent risk marker for any form of diabetes mellitus, and a value ≥50% alleviates the risk of developing T2DM by two to three times [35]. Another study has demonstrated the presence of three to four times higher values of transferrin saturation (>35%) in T2DM patients, compared to the documented values in the general population [36]. Such findings have linked elevated transferrin saturation in T2DM patients with earlier age of onset, and our results reflect the same.

Linear relationships between free iron and glycated haemoglobin have been shown in in vitro experiments [37]. It has been shown that H_2_O_2_ invokes the release of iron, far more from glycosylated haemoglobin than that from the non-glycosylated form. Similarly, arachidonic acid and deoxyribose in the presence of H_2_O_2_ are degraded in a far better way by HbA1c than by non-glycated haemoglobin (HbA0), giving reason to believe that iron release is stupendous with HbA1c as compared to HbA0. On the contrary, the peroxidase activity of HbA1c is less than that of HbA0. Such reactions involving haemoglobin point towards a system of the copious generation of free radicals and oxidative stress in T2DM [38].The results of our study showed that serum iron concentration in T2DM patients with suboptimal glycaemic control (Group III) is significantly elevated compared to controls, but it did not show a significant correlation with Tsat or HbA1c values in the patients of Group III. The absence of long-standing diabetic co-morbidities in our T2DM patients may be the reason behind this. When present, these play a crucial role in the vicious cycle of hyperglycaemia and subsequent metabolic distortion [39,40,41]. Such variation has also been observed in a survey-based study where the level of serum ferritin (index for body iron stores) showed no correlation with blood sugar and HbA1c in diabetic patients [42].

Elevated serum iron concentration among the general population is found in cases of haemolytic anaemia, hepatitis, and lead and iron poisoning, whereas low serum iron concentration is a marked feature of anaemia caused by iron deficiency due to the impaired intake or absorption of iron, heavy blood loss, late pregnancy, and cancer. The role of iron in the pathogenesis of T2DM calls for further studies owing to increased incidence of iron overload encountered among diabetics, which can be reversed by achieving targets of good glycaemic control using either phlebotomy or iron chelation therapy [43].

An increase in the levels of serum free iron concentration and serum transferrin saturation levels with poor glycaemic control in our study indicate an important role of free iron in the development of diabetic complications. A study in Iran has pointed out that elevated levels of iron in first-degree relatives of T2DM patients might be a predisposing factor for them towards the development of diabetes in future or vice versa (i.e., as a result of diabetes development) [32]. Knowledge and awareness about diabetic complications among the affected individuals and their family is the need of the hour to postpone their onset and progression. Thus, monitoring the prevalence of iron overload is beneficial in the long run.

## 5. Conclusions

Hence, the study of iron and related parameters can be a useful offshoot of the conventional studies on diabetes and its complications. Iron overload associated with poor glycaemic control can thus be harnessed as a valuable marker for diabetes pathogenesis in not only T2DM patients, but also their first-degree relatives. Similar studies with larger cohort size including patients with co-morbidities will further present an expanded view of the current situation.

## Figures and Tables

**Table 1:** General features of the subjects.

Parameter	Group I	*p* Value (I vs. II)	Group II	*p* Value (II vs. III)	Group III	*p* Value (I vs. III)
Number	50	-	50	-	50	-
Male/Female	35/15	-	36/14	-	35/15	-
Age*	48.78 ± 1.647	0.2137	52.4 ± 1.377	0.9154	53.26 ± 1.517	0.959
Disease duration (years)	-	-	<7	-	>7	-
FPG (mg/dL)*	110.1 ± 2.231	<0.0001	182 ± 8.947	0.9448	185.4 ± 9.131	<0.0001
HbA1c (%)*	6.58 ± 0.069	0.0001	7.21 ± 0.066	0.000	8.74 ± 0.103	0.0001
BMI (kg/m^2^)*	25.15 ± 0.43	0.0007	27.33 ± 0.42	0.826	26.99 ± 0.37	0.0051

*Values expressed in mean ± standard error of the mean (SEM). Group I: Healthy controls; Group II: Type 2 diabetes mellitus (T2DM) patients with optimal glycaemic control; Group III: T2DM patients with suboptimal glycaemic control. BMI: body mass index; HbA1c: glycated haemoglobin; FPG: fasting plasma glucose.

**Table 2:** Comparison of iron profile between Group I (healthy controls), Group II (T2DM cases with good glycaemic control), and Group III (T2DM cases with poor glycaemic control)*.

Parameter	Group I	*p* Value (I vs. II)	Group II	*p* Value (II vs. III)	Group III	*p* Value (I vs. III)
Serum iron (µg/dL)	105.34 ± 3.5	0.9178	107.33 ± 3.45	0.0012	125.58 ± 3.74	0.0003
TIBC (µg/dL)	311.39 ± 5.47	0.9674	309.63 ± 6.1	0.0015	284.2 ± 3.18	0.0006
Tsat (%)	34.17 ± 1.21	0.8622	35.02 ± 1.2	0.0002	44.39 ± 1.07	0.0001

*Values expressed as mean ± SEM.TIBC: total iron binding capacity; Tsat: transferrin saturation.

**Table 3:** Correlation of serum iron concentration with HbA1c and Tsat in Group III cases.

Parameter	Pearson Coefficient (*r*)	*p*Value
HbA1c	0.05	0.73
Tsat	0.0496	0.732
